# Vicrostatin – An Anti-Invasive Multi-Integrin Targeting Chimeric Disintegrin with Tumor Anti-Angiogenic and Pro-Apoptotic Activities

**DOI:** 10.1371/journal.pone.0010929

**Published:** 2010-06-03

**Authors:** Radu O. Minea, Corey M. Helchowski, Samuel J. Zidovetzki, Fritz K. Costa, Stephen D. Swenson, Francis S. Markland

**Affiliations:** Department of Biochemistry and Molecular Biology and Norris Comprehensive Cancer Center, Keck School of Medicine, University of Southern California, Los Angeles, California, United States of America; Griffith University, Australia

## Abstract

Similar to other integrin-targeting strategies, disintegrins have previously shown good efficacy in animal cancer models with favorable pharmacological attributes and translational potential. Nonetheless, these polypeptides are notoriously difficult to produce recombinantly due to their particular structure requiring the correct pairing of multiple disulfide bonds for biological activity. Here, we show that a sequence-engineered disintegrin (called vicrostatin or VCN) can be reliably produced in large scale amounts directly in the oxidative cytoplasm of Origami B *E. coli*. Through multiple integrin ligation (i.e., αvβ3, αvβ5, and α5β1), VCN targets both endothelial and cancer cells significantly inhibiting their motility through a reconstituted basement membrane. Interestingly, in a manner distinct from other integrin ligands but reminiscent of some ECM-derived endogenous anti-angiogenic fragments previously described in the literature, VCN profoundly disrupts the actin cytoskeleton of endothelial cells (EC) inducing a rapid disassembly of stress fibers and actin reorganization, ultimately interfering with EC's ability to invade and form tubes (tubulogenesis). Moreover, here we show for the first time that the addition of a disintegrin to tubulogenic EC sandwiched *in vitro* between two Matrigel layers negatively impacts their survival despite the presence of abundant haptotactic cues. A liposomal formulation of VCN (LVCN) was further evaluated *in vivo* in two animal cancer models with different growth characteristics. Our data demonstrate that LVCN is well tolerated while exerting a significant delay in tumor growth and an increase in the survival of treated animals. These results can be partially explained by potent tumor anti-angiogenic and pro-apoptotic effects induced by LVCN.

## Introduction

Despite the tremendous progress made in the last decades in deciphering the molecular intricacies of various signaling circuitries that operate aberrantly in cancers and the therapeutic advancement seen with some of the newer anti-cancer modalities recently approved by the FDA, such as humanized monoclonal antibodies directed at VEGF-A (vascular endothelial growth factor A) and receptor tyrosine kinases [1], [2] or non-peptide tyrosine kinase inhibitors [3], [4], the 5-year prognosis for most solid tumors remains reserved. Consequently, there is still a significant need to identify new drug candidates with broader spectrums of activity directed at signaling platforms (regulatory molecular hubs) shared by distinct cancer processes, which are, thus, able to simultaneously target multiple pathological aspects of cancer (for instance, both tumor angiogenesis and metastasis) with fewer side effects. The ability of transformed cells to evade the restrictive environmental control exerted by the normal tissue architecture and grow in an anchorage-independent fashion is one of cancer's hallmarks [5]. One class of cell-surface receptors known to play a critical role in the process leading to the acquisition of an anchorage-independent phenotype is represented by the integrins [6].

Integrins are heterodimeric receptors that evolved to mediate the complex cell-ECM interactions that regulate the ability of cells to mechanically sense their environment by assembling complex multimolecular platforms capable of integrating multiple signaling pathways initiated by extracellular cues with the cellular cytoskeleton. In the ecology of multicelular organisms integrins are major contributors to the homeostasis of tissue architecture by keeping epithelial cells in a differentiated, specialized state [7]. Conversely, as epithelia transition to malignancy they evade the microenvironmental constraints by both altering their integrin affinity and avidity for ECM proteins (inside-out signaling) and/or shifting their integrin expression [6], [8]. The precise roles, however, played by different integrin subunits in various aspects of tumor progression and why some integrins appear to be especially supportive of tumor progression [9] are still not fully understood. Despite these limitations, due to their pivotal roles in cancer biology, integrins represent attractive therapeutic targets. For instance, although it doesn't seem to be essential for the formation of vasculature during development [10], nor during physiological angiogenesis associated with wound healing or tissue repair [11], [12], the β3 integrin appears to be critically involved in the regulation of pathological angiogenesis [13]. Therefore, the pharmacological blockade of the β3 integrin has been demonstrated to significantly reduce tumor angiogenesis in numerous cancer models, a finding that has eventually led to the development of several drug candidates currently in clinical trials [14], [15]. Similarly, αvβ5 and α5β1 as well as a number of other integrins (notably α2β1, α4β1, and α6β4) have also been shown to play important roles in tumor angiogenesis, their pharmacological targeting by soluble ligands or monoclonal antibodies leading to reduced tumor microvessel density in various cancer models [12], [16]. Furthermore, at least some of the complex effects elicited by several endogenous ECM-derived antiangiogenic fragments (e.g., endostatin, tumstatin, endorepellin, etc) are attributed to direct integrin engagement [17], [18]. In this report, we provide further evidence in support of the above therapeutic paradigm by showing that the efficient disruption by a member of the disintegrin family of multiple integrin pathways upregulated in cancer is followed by significant tumor anti-angiogenic and pro-apoptotic effects.

Disintegrins are among the most potent soluble ligands of integrins representing a class of cysteine-rich polypeptides historically isolated from the venoms of snakes belonging to the *Viperidae* family [19]. These small polypeptides hold a significant translational potential as anti-cancer agents based on their anti-angiogenic and anti-metastatic effects demonstrated in various experimental settings [20], [21], [22]. The integrin-binding activity of disintegrins depends on the appropriate pairing of several cysteine residues responsible for the disintegrin fold, a mobile 11-amino acid loop protruding from the polypeptide core displaying a tri-peptide motif, usually RGD (Arg-Gly-Asp), that is conserved in many disintegrins [23], [24]. Although these molecules naturally evolved to efficiently bind to the activated platelet-specific integrin αIIbβ3, thus disrupting the process of platelet aggregation (the final step in blood clotting), most purified snake venom disintegrins are rather promiscuous in that they bind to several β1, β3 or β5 integrin members, albeit with different affinities and selectivity [25]. Two of the most studied native disintegrins are the homodimeric contortrostatin (CN) [26] and the monomeric echistatin [20]. Similar to echistatin, the anti-tumor activity of CN is based on its high affinity interaction with integrins α5β1, αvβ3 and αvβ5 on both cancer and angiogenic endothelial cells [27], [28], [29]. In a previous study [22] we showed that a liposomal formulation of CN limited tumor growth and significantly reduced microvascular density in a xenograft animal cancer model. We provided evidence that CN can be safely and effectively administered intravenously by a clinically acceptable delivery method (i.e., liposomal delivery) and, by doing so, CN passively accumulates at the tumor site. Furthermore, liposomal CN did not interact with the components of blood coagulation system nor elicit a neutralizing antibody immune response. For eventual clinical use, however, the direct isolation of native CN from crude venom would be laborious and prohibitively expensive since this polypeptide only exists as a very minor fraction relative to other venom components.

Vicrostatin (VCN) is a chimeric disintegrin generated recombinantly by grafting the C-terminal tail of **vi**perid snake venom disintegrin echistatin to the sequence of **cro**talid disintegrin contortrostatin (CN); we have previously shown [30] that this novel sequence could be produced as an active polypeptide in Origami B *E. coli.* Here, we show that VCN retains the binding profile of CN yet it engages integrins in a unique manner. As previously shown with native CN, VCN also appears to engage integrins agonistically thus behaving like a soluble ECM-mimetic. Via agonistic integrin ligation in the absence of tethering, VCN appears to inappropriately elicit a cascade of signaling events rapidly leading to actin stress fibers disassembly in HUVEC (human umbilical vein endothelial cells) plated on complete Matrigel. Moreover, in a manner reminiscent of some ECM-derived endogenous anti-angiogenic fragments [31], [32], [33], VCN interferes with the assembly of a dynamic actin cytoskeleton in tubulogenic HUVEC sandwiched between two Matrigel layers, negatively impacting the survival of these cells. Finally, in an effort to address our main goal of developing an efficient and clinically relevant delivery method for recombinant disintegrins, VCN was packaged in a liposomal formulation (LVCN) and further evaluated for *in vivo* efficacy.

## Materials and Methods

### Ethics Statement

All animals involved in this study were handled and euthanized in strict accordance with good animal practice as defined by the strict guidelines of the Institutional Animal Care and Use Committee (IACUC) of the University of Southern California.

### Controtrostatin purification

Venom of *Agkistrodon contortrix contortrix* was purchased from Miami Serpentarium (Punta Gorda, FL). CN was purified in a four-step high-performance liquid chromatography (HPLC) procedure according to an established protocol [26].

### Cells and reagents

The MDA-MB-435 cells were obtained from Dr. Janet Price (MD Anderson Cancer Center, Houston, TX) and the MDA-MB-231 cells from Dr. Toshiyuki Yoneda (Osaka University, Osaka, Japan). HUVEC were purchased from PromoCell (Heidelberg, Germany) and maintained according to the manufacturer's protocol. The Origami B (DE3) *E. coli* strain and pET32a expression vector carrying the bacterial thioredoxin A gene (*trxA*) were purchased from Novagen (San Diego, CA). The oligonucleotide primers used for rCN and VCN cloning were synthesized by Operon Biotechnologies, Inc. (Huntsville, AL). A southern copperhead venom gland cDNA library, a mouse CN monoclonal antibody, and rabbit CN polyclonal antiserum (Alpha Diagnostic Intl., San Antonio, TX) are available in the Markland laboratory at the University of Southern California. ‘Endothelial Cell Tube Formation’ plates were purchased from BD Biosciences (Bedford, MA). The tube formation inhibitor Suramin, the actin modifier Cytochalasin D (CytoD), and the cyclo(Arg-Gly-Asp-DPhe-Val) peptide (cRGDfV) were purchased from Calbiochem (San Diego, CA). The fluorometric cell invasion assay kit (QCM™ 24-Well Cell Invasion) was from Millipore (Billerica, MA). The complete Matrigel was from BD Biosciences. Recombinant tobacco etch virus (TEV) protease, Calcein AM, and Rhodamine-Phalloidin were purchased from Invitrogen (Carlsbad, CA). A column-based fluorescein isothiocyanate (FITC)-labeling kit (EZ-Label) and an endotoxin removal kit were purchased from Pierce (Rockford, IL). The DeadEnd™ Fluorometric TUNEL (terminal deoxynucleotidyl transferase dUTP nick end labeling) assay kit was from Promega (Madison, WI). The non-selective protein kinase inhibitor Staurosporine (STSP) was from Cayman Chemical (Ann Arbor, MI). The murine β3 integrin 7E3 antibody was a gift from Dr. Marian Nakata (Centocor, Horsham, PA). The murine αvβ3 integrin antibody LM609 was from Millipore. The CD31 polyclonal antibody (MEC13.3) was from BD Pharmingen (Franklin Lakes, NJ). The Ki-67 (H-300), a focal adhesion kinase (FAK) polyclonal (A-17), and all secondary antibodies were purchased from Santa Cruz Biotechnology (Santa Cruz, CA). A FAK monoclonal antibody (clone 77) was from BD Biosciences. A phosphotyrosine monoclonal antibody (P-Tyr-102) was from Cell Signaling Technology (Danvers, MA). Purified soluble αvβ3 and αvβ5 integrins were purchased from Millipore and soluble recombinant α5β1 integrin from R&D Systems (Minneapolis, MN). All other reagents were purchased from Sigma Chemical Co. (St. Louis, MO). Avastin was a gift from Dr. Agustin Garcia (Norris Comprehensive Cancer Center, University of Southern California).

### Construction of rCN and VCN expression vectors and recombinant production

rCN and VCN were cloned into pET32a vector downstream of thioredoxin A (TrxA) using a BglII/NcoI set of restriction enzymes. The forward primers for both rCN and VCN introduced a unique TEV protease cleavage site, which made possible the removal of thioredoxin during purification. To build the VCN construct, the nucleotides encoding the C-terminal tail of echistatin were added to CN via an elongated reverse primer. The primers used for rCN were: forward - 5′gttccagatctcgagaatctttacttccaaggagacgctcctgcaaatccgtgctgcga3′, and reverse - 5′gttattcgccatggcttaggcatggaagggatttctgggacagccagcaga3′. The primers used for VCN were: forward - 5′gttccagatctcgagaatctttacttccaaggagacgctcctgcaaatccgtgctgcga3′, and reverse - 5′gttattcgccatggcttaagtagctggacccttgtggggatttctgggacagccagcagatatgcc3′. Both plasmids were initially amplified in DH5α *E. coli*, purified and sequenced, and then transferred into Origami B (DE3) *E. coli*. Multiple cultures were established for each construct from individual colonies of transformed BL21 (DE3), AD494 (DE3) or Origami B (DE3) in LB media containing either carbenicillin (50 µg/mL) alone, or carbenicillin (50 µg/mL) plus kanamycin (15 µg/mL) or carbenicillin (50 µg/mL) plus tetracycline (12.5 µg/mL), plus kanamycin (15 µg/mL) and grown at 37°C and 250 rpm in a shaker-incubator until they reached an OD_600_ of 0.6-1. At this point, the cells were induced in 1 mM IPTG (isopropyl-1-β-D-thio-1-galactopyranoside) and incubated for another 4–5 hours at 37°C and 250 rpm. At the end of the induction period, the cells were pelleted at 4000×g and lysed in a microfluidizer (Microfluidics M-110L, Microfluidics, Newton, MA). The operating conditions of the microfluidizer included applied pressures of 14,000–18,000 psi, bacterial slurry flow rates of 300–400 ml per minute and multiple passes of the slurry through the processor. The lysate insoluble cellular debris was removed by centrifugation (40,000×g) and the soluble material containing either Trx-rCN or Trx-VCN collected. The expressed fusion proteins in the collected soluble lysates were then proteolysed by incubation with recombinant TEV protease overnight at room temperature which efficiently cleaved off rCN or VCN from TrxA as monitored by SDS-PAGE (sodium dodecyl sulfate-polyacrylamide gel electrophoresis). When proteolysis was complete, the proteolyzed lysates were passed through a 0.22 µm filter, diluted 1∶100 in ddH_2_O, ultrafiltrated through a 50,000 MWCO cartridge (Biomax50, Millipore) and then reconcentrated against a 5,000 MWCO cartridge (Biomax5, Millipore) using a tangential flow ultrafiltration device (Labscale TFF system, Millipore).

### Purification of recombinant disintegrins

This was done by C18-reverse phase HPLC using the standard elution conditions previously employed for the purification of native CN [26]. The filtrated lysates processed as described above were loaded onto a Vydac C18 column (218TP54, Temecula, CA). A ten-minute rinse (at 5 ml/min) of the column with an aqueous solution containing 0.1%TFA was followed by a linear gradient (0–100%) elution over 150 min in a mobile phase containing 80% acetonitrile and 0.1%TFA. rCN starts eluting in 30% acetonitrile, while VCN elutes in 35% acetonitrile.

### Inhibition of platelet aggregation

The inhibition of ADP-induced platelet aggregation by recombinant disintegrins was determined by measuring the light absorption of human platelet-rich plasma (PRP) in a specialized spectrophotometer (Chrono-log 490 optical aggregometer, Chrono-log, Havertown, PA) as previously described [22]. The FITC-labeled disintegrins (FITC-CN and FITC-VCN) and the liposomal formulations of VCN were also tested for activity against platelets.

### Mass spectrometry (MS) analysis and sequencing by tryptic digestion

The MS analysis (MALDI-TOF and ESI) was initially done by Dr. Kym Faull (University of California at Los Angeles) and the subsequent sequencing by Dr. Ebrahim Zandi (Keck School of Medicine, University of Southern California). For sequencing, the purified recombinant disintegrins were reduced, alkylated and digested with trypsin at 37°C overnight. The resultant digestion peptides were then used in the tandem LC/MS/MS for sequence analysis. The LC consists of a reverse phase C-18 column through which peptides were eluted into the mass spectrometer using the following gradients: 5–60% acetonitrile +0.1% formic acid over 75 min and 50–90% acetonitrile +0.1% formic acid over 10 min. Tandem MS/MS spectra was acquired with Xcalibur software on a linear ion trap LTQ instrument. Data was analyzed using Bioworks, the SEQUEST algorithm and Sage-N Sorcerer to determine cross-correlation scores between acquired spectra and NCBI protein FASTA databases or any other databases as needed.

### FAK phosphorylation studies

Serum-starved MDA-MB-435 cells were harvested by limited trypsin/EDTA treatment [34] and maintained in suspension before being exposed for 10–30 min to different concentrations of either native CN or VCN. The cells were lysed and the soluble fraction immunoprecipitated with a polyclonal FAK antibody (clone A-17) and further assayed by Western blotting [34], [35]. The transferred proteins were probed with either a p-Tyr antibody (P-Tyr-102, Cell Signaling Technology, Danvers, MA) or a monoclonal FAK antibody (clone 77).

### Cell surface binding studies by flow cytometry

HUVEC, MDA-MB-231 or MDA-MB-435 cells were grown to early confluency and starved overnight in serum-free media. The cells were harvested and resuspended in 1 ml of serum-free media (5×10^5^ cells/condition) before being incubated with different treatments or controls for 30 min at 37°C. At the end of the incubation period, the cells were pelleted, washed in ice-cold PBS containing 5% fetal bovine serum and either analyzed in a FACSCalibur scanner or, depending on the assay, further incubated at 4°C for 30 min intervals with additional treatments. All cells were counterstained with propidium iodide to allow gating of necrotic cells. For each reading, 10,000 cells per sample were analyzed.

### Integrin binding kinetics by fluorescence polarization (FP)

Differing concentrations of purified soluble functional integrins (i.e., αvβ3, αvβ5 or α5β1) were incubated with a constant amount of FITC-labeled VCN or CN using an established protocol [36]. Upon binding to the much larger integrin, the fluorescent tag on either disintegrin tumbles in solution at a slower rate compared to the unbound state resulting in increased levels of polarization. The measured FP value is a weighted average of FP values of the bound and free fluorescent disintegrins and is therefore a direct measure of the bound fraction. The data were analyzed as for standard radioligand binding, and kinetics of binding determined using Scatchard analysis and a non-linear curve fit. The data were generated in a PTI QuantaMaster QM-4SE spectrofluorometer (Photon Technology International, Birmingham, NJ) using the PTI FeliX32 software for data acquisition and Prism v3.02 (GraphPad Software, La Jolla, CA) for data analysis.

### Cell viability studies

HUVEC, MDA-MB-231 or MDA-MB-435 cells were plated in serum-free media on Matrigel-coated multi-well glass chamber slides (5×10^4^ cells/well) and allowed to adhere. Native CN or VCN were added to the wells at concentrations ranging from 1–1000 nM. Cells receiving no treatment or a known apoptosis inducer (Staurosporine) were used as controls. The cell viability for each condition was assessed after incubation times up to 48 hr both colorimetrically using the Cell Titer 96 AQueous cell viability kit (Promega, Madison, WI) according to the manufacturer's protocol and by TUNEL staining. For TUNEL, the cells were stained using the DeadEnd™ Fluorometric kit (Promega, Madison, WI), and then counterstained with Hoechst 33342. The TUNEL- and Hoechst-stained areas were quantitated digitally by pixel counting on images taken from multiple slides per condition using the ‘SimplePCI’ imaging software (Hamamatsu Corporation, Sewickley, PA).

### Inhibition of cell invasion

The ability of disintegrins to block the invasion of HUVEC, MDA-MB-231 or MDA-MB-435 cells through a reconstituted basement membrane was assessed using the fluorometric QCM™ 24-Well Cell Invasion kit (Millipore, Billerica, MA). The cells were serum-starved overnight, harvested, resuspended in serum-free media (1×10^6^ cell/ml) and incubated in the presence of various concentrations (0–1000 nM) of either native CN or VCN for 10 min at 37°C. The assay was done according to the manufacturer's protocol and used HT1080 conditioned media as a chemoattractant. The invasion plates were incubated for up to 48 hr (depending on the cell line) at 37°C in the presence of 5% CO_2_. At the end of the incubation period, the invaded cells were detached, lysed and quantitated using the DNA-binding fluorescent dye CyQUANT. The relative fluorescence was measured in a SPECTRAmax GeminiEM fluorescent plate reader (Molecular Devices, Sunnyvale, CA) and the numbers averaged and plotted for each condition.

### Inhibition of HUVEC tube formation

‘Endothelial Tube Formation’ plates precoated with Matrigel (BD Biosciences, Bedford, MA) were used according to the manufacturer's protocol. HUVEC were seeded in triplicate (3×10^4^ cells/well) in the presence of various concentrations (0–1000 nM) of either native CN or VCN and incubated for 16 hr at 37°C in the presence of 5%CO_2_. The tube formation inhibitor Suramin was used as a positive control. At the end of incubation period, cells were stained with Calcein AM and imaged by confocal microscopy (LSM 510 Confocal/Titanium Sapphire Laser). The total length of tubes for each condition was quantitated in multiple fields using the Zeiss LSM Image Browser (Carl Zeiss MicroImaging GmbH, Munich, Germany) and averaged from at least three independent experiments.

### Disruption of actin cytoskeleton organization

HUVEC grown in complete media were seeded in triplicate in 8-well chamber slides coated with complete Matrigel (4×10^4^ cells/well). Each well received different concentrations of various treatments {including FITC-CN, FITC-VCN, the cyclic RGD peptide cyclo(Arg-Gly-Asp-DPhe-Val) (abbreviated cRGDfV), the murine 7E3 or LM609 monoclonal antibodies}. The actin modifier Cytochalasin D (CytoD) was used as a positive control. The cells were incubated with the treatments for 3 hr at 37°C in the presence of 5%CO_2_. At the end of the incubation period, the cells were washed, incubated with secondary treatments (depending on the condition), fixed in 4% formaldehyde, permeabilized in 0.1% Triton X-100 in PBS, and then stained with Rhodamine-Phalloidin and counter-stained with Hoechst 33342 before being imaged by confocal microscopy (LSM 510 Confocal/Titanium Sapphire Laser).

### *In vitro* Matrigel-embedded HUVEC apoptosis studies

HUVEC seeded in serum-free media in 8-well chamber slides coated with complete Matrigel (4×10^4^ cells/well) were allowed to adhere before being sandwiched with another layer of Matrigel that was uniformly pipetted on top of the adherent cells. The second Matrigel layer was allowed to settle before various treatments were added and chambers incubated at 37°C in the presence of 5%CO_2_ for approximately 16 hr. At the end of the incubation period, the cells were fixed in 4% formaldehyde, permeabilized in 0.2% Triton X-100 in PBS, TUNEL stained using the DeadEnd™ Fluorometric kit (Promega, Madison, WI), and counterstained with Rhodamine-Phalloidin and Hoechst 33342. The % cell death was quantitated in random fields taken at ×250 magnification using the formula ‘number of TUNEL^+^ nuclei/total number of nuclei x100’ for each treatment group. The TUNEL- and Hoechst-stained areas were quantitated digitally by pixel counting on images taken in random fields from multiple slides per condition using the ‘SimplePCI’ imaging software (Hamamatsu Corporation, Sewickley, PA).

### Liposomal encapsulation of VCN

This procedure was carried out by Molecular Express, Inc (Los Angeles, CA) a company specializing in liposomal encapsulation of therapeutic proteins and other drugs. Briefly, stock solutions of phospholipids and cholesterol were prepared by dissolving each lipid in a chloroform/methanol solvent mixture. Thin lipid films were created by pipetting aliquots of the lipid solutions into round bottom glass tubes followed by solvent evaporation at 65°C under a stream of nitrogen gas with the lipids and cholesterol further dried under vacuum for 48 hours. Dried VCN was then dissolved in a hydration buffer (10 mM sodium phosphate and 262 mM sucrose, pH 7.2) and added to the dried lipids. After 5 min incubation at 50°C, liposomal VCN (LVCN) particles were generated by either probe sonication at 10% power for 3 to 5 min in a Branson Probe Sonifier or homogenized in a microfluidizer (M110L; Microfluidics, Newton, MA). The homogenized material was processed between 10,000 and 18,000 psi while maintaining an elevated temperature (45–65°C). Samples from each batch were taken during the process and the size distribution of LVCN was determined with an Ultrafine Particle Analyzer (UPA150; Microtrac, North Largo, FL). After processing, the unencapsulated VCN in each batch was removed by ultrafiltration using an Amicon UF membrane of 100,000 MWCO and the LVCN was further sterilized by filtration through a 0.2 µM PVDF filter.

### *In vivo* efficacy studies

MDA-MB-435 cells (5×10^5^ per inoculum) or MDA-MB-231 cells (2×10^6^ per inoculum) were harvested and resuspended in complete Matrigel and injected in the mammary fat pads of nude mice as previously described [22]. The tumors were allowed to grow for 2 weeks or until they became palpable before treatment was initiated. VCN was administered either encapsulated in different liposomal formulations (at the dose-equivalent of 100 µg of dry VCN per injection) or non-encapsulated, as an aqueous solution (100 µg VCN). All VCN administrations were made intravenously (via tail vein) twice a week for the duration of each study. Avastin was administered intravenously (via tail vein) at the dose of 400 µg per injection (approx. 20 µg/gr.) once a week for the duration of the MDA-MB-231 study. Tumor diameters were measured weekly with a caliper in a blind fashion and the tumor volumes calculated using the formula [length (mm) × width (mm)^2^]/2, where the width and the length were the shortest and longest diameters, respectively [37]. The average tumor volume for each study group was plotted as a function of time and type of treatment during the entire course of each study.

### Tumor microvessel quantitation

Acetone-fixed 5 µm-thick cryostat sections cut from MDA-MB-231 tumors were air dried for 30 min before being blocked in 5% BSA in PBS and incubated overnight with a rat polyclonal CD31 antibody (clone MEC13.3) at room temperature. The working dilution for this antibody was 1/100 in 5%BSA in PBS. After washing off the unbound CD31 antibody, a biotinylated goat anti-rat antibody (1/150 in 5%BSA in PBS) was applied for 45 min at room temperature followed by the Avidin Binding Complex (Vector Laboratories, Burlingame, CA) for another 30 min and the addition of 3-amino-9-ethylcarbazole chromogen (a peroxidase substrate). The slides were counterstained with hematoxylin. To quantitate the CD31-stained microvessels, the slides were subjected to ‘random field’ analysis [38], [39]. Random field images were captured at ×200 (10 images were analyzed per tumor and 4 random tumors were analyzed from each animal group). The CD31-positive areas were quantitated for each random field as % of total stained area by pixel counting using the ‘SimplePCI’ imaging software (Hamamatsu Corporation, Sewickley, PA). To eliminate bias, the random field image capture and the subsequent processing and analysis were carried out in a blind fashion.

### TUNEL and Ki-67 staining of tumor sections

For tumor apoptosis, the DeadEnd™ Fluorometric TUNEL assay kit (Promega, Madison, WI) was used according to the manufacturer's protocol. Importantly, we found that the TUNEL staining was optimal only when the cryostat sections were fixed and permeabilized in a cold ethanol/acetic acid (2/1) bath for 5 min at −20°C. Any other fixation and/or permeabilization technique yielded suboptimal staining results. However, this above approach is not compatible with CD31 antibody staining, which works best on either not-fixed or acetone-fixed tissues. After TUNEL staining, the nuclei were counterstained with Hoechst 33342. The nuclei from apoptosis ‘hotspot’ areas were digitally counted (object counting) using the SimplePCI imaging software on random images (at least 10 images per tumor from multiple tumors per group) captured at ×250. The % of cell death was plotted using the formula ‘number of TUNEL^+^ nuclei/total number of nuclei x100’ for each treatment group. For Ki-67 staining, a primary rabbit antibody (clone H-300) and a biotinylated anti-rabbit secondary were used. The sections were acetone-fixed, incubated with the antibodies and prepared for immunoperoxidase staining as described above for CD31 staining. The Ki-67^+^ nuclei were quantitated by pixel counting as % of total stained area as described above for CD31.

### Statistical analysis

Statistical significance was analyzed in Prism v.3.2 (GraphPad Software, La Jolla, CA) by unpaired t-test followed by F-test to compare variances. The tumor volume distribution and immunohistochemistry data were assessed by analysis of variance (ANOVA) with a significant overall F-test followed by Dunnett's multiple comparison tests of treatment groups relative to control. Two-tailed *P*<0.05 were considered significant.

## Results

### The recombinant production of VCN as an active soluble protein

The expression of recombinant disintegrins was done in Origami B (DE3), a strain uniquely designed to address the shortcomings of disulfide-rich recombinant protein production in wild-type *E. coli*. This strain is engineered to produce defective forms of two redox enzymes that are critically involved in controlling the major reductive pathways in this bacterium: thioredoxin reductase (TrxB) and glutathione reductase (Gor). In the absence of functional cytoplasmic TrxB and Gor enzymes, the redox equilibrium in this *E. coli* strain is shifted towards oxidation, a redox state that greatly promotes disulfide bond formation in heterologous proteins expressed in this compartment [40].

The Origami B strain is better suited for recombinant protein expression than most other commercially available *E. coli* strains since, in addition to providing a favorable environment for disulfide bond formation, by being a derivative of the BL21 strain it is also deficient in *ompT* and *lon* proteases. Moreover, this strain is lac permease (*lacY1*) deficient, a feature that enables a uniform entry of IPTG (a lactose derivative commonly employed to trigger the expression of recombinant proteins engineered downstream of a *lac* promoter) into cells with a more homogenous level of induction and, consequently, adjustable levels of protein expression.

The pET32a expression vector and the T7 system are designed for robust expression of heterologous proteins fused to the 109 amino acid bacterial thioredoxin A (TrxA) in DE3 lysogens. In wild-type *E. coli*, TrxA normally functions as a major cytoplasmic reductase under tight regulatory control. However, in the Origami strain, the oxidative redox state perpetuated by defective TrxB and Gor enzymes ‘tricks’ this bacterium into producing compensatory higher amounts of TrxA reductase in an attempt to restore the wild-type redox equilibrium which in turn drives the robust expression of any recombinant protein genetically fused to TrxA. Another advantage of expressing heterologous proteins fused to TrxA is the high solubility of this bacterial protein, the result of which is that TrxA internally chaperones the recombinant protein fused to it thus keeping it in solution and allowing for higher levels of foreign protein accumulation in the cytoplasm [40].

**Figure 1:**
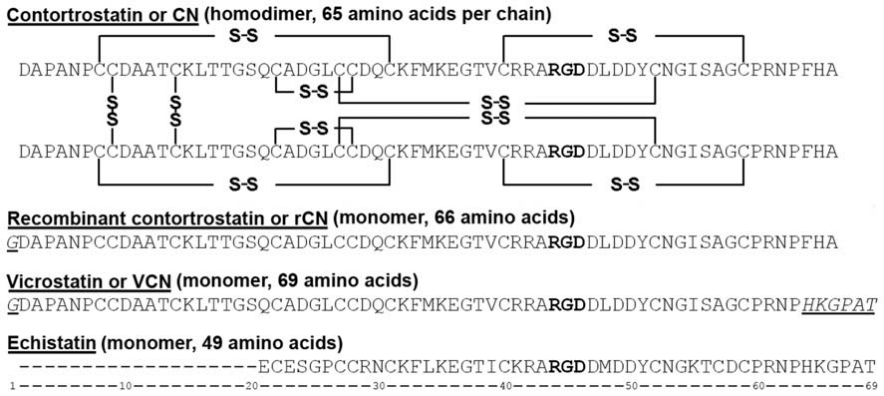
Comparison between contortrostatin (CN), recombinant contortrostatin (rCN), vicrostatin (VCN), and echistatin sequences. Mass spectrometry and crystallographic data have confirmed that CN is a dimer with two identical chains oriented in an antiparallel fashion and held together by two interchain disulfide bonds. Unlike native CN, mass spectrometry showed that both rCN and VCN are monomers. In the above sequences, the Arg-Gly-Asp tripeptide motif is depicted in bold whereas the non-native amino acids in rCN and VCN are both italicized and underlined.

To explore the recombinant production of disintegrins in Origami B (DE3) we generated two constructs: one based on the exact sequence of native CN (referred to as rCN) and a chimeric construct, previously designated as rCN+ [30], but now referred to as vicrostatin (VCN). The VCN construct was designed by replacing the C-terminal tail of native CN with the tail of another native disintegrin, echistatin, a short length viperid disintegrin. The sequence alignment of CN, rCN, VCN and echistatin (also known as echistatin alpha) [20] are shown in **Fig. 1**. The rationale for the VCN design was based on the finding that the C-termini of snake venom disintegrins are important structural elements essential for full disintegrin activity, and have been shown to participate in the ligation of the receptor together with the disintegrin loop [41]. In addition, a previous report [42] had demonstrated that the swapping of C-terminal tails between two native disintegrins may actually lead to the generation of novel chimeric molecules capable of recognizing specific integrins with altered binding affinities. Our expectation for VCN was that, by carrying a modified C-terminus, it would display an improved affinity compared to the native molecule for integrin α5β1, a major player in angiogenesis. The participation of multiple regions in disintegrins in receptor binding emphasizes the complexity of these bigger polypeptides compared to small cyclic RGD peptides or RGD-peptidomimetics. Moreover, these structural differences are also expected to be reflected at the functional level, and in this report we provide the evidence that this is the case.

**Figure 2:**
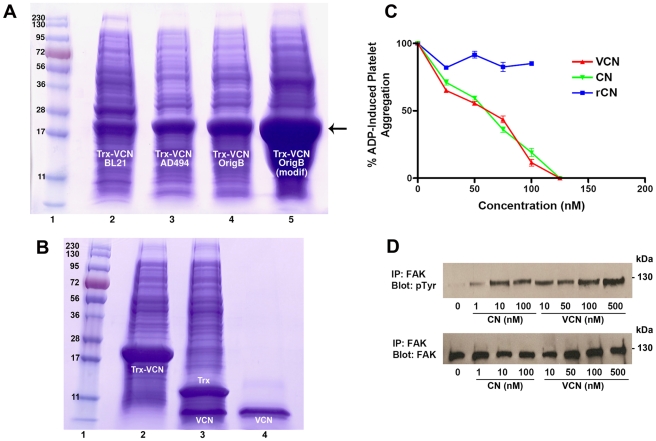
The expression, purification and initial characterization of VCN as an active disintegrin. **Panel A –** The production of Trx-VCN was assessed in different *E. coli* strains that were transformed, grown, induced, and processed under identical conditions. The same amount (5 µl) of cell lysates from each induced strain was loaded on a precast gel and Coomassie stained. Unlike the BL21 (DE3) strain, the lysates from both AD494 (DE3) and Origami B (DE3) strains generate a unique and consistent Trx-VCN band (shown by the arrow). By employing a modified media recipe, the Origami B (DE3) Trx-VCN transformants achieve higher cell densities at the end of the induction time, generating up to 200 mg of soluble VCN per L of bacterial culture after purification. **Panel B –** Coomassie stained gel showing the migration of Trx-VCN before and after TEV proteolysis (lanes 2 and 3, respectively) versus C18 reverse phase-HPLC purified VCN (lane 4). **Panel C** **–** VCN and native CN exhibit an almost identical dose-dependent inhibitory effect against ADP-induced platelet aggregation when incubated with human platelet-rich plasma (with a calculated IC_50_ of ∼60 nM). In contrast, the rCN construct, which is also expressed as a soluble polypeptide in Origami B (DE3), shows no inhibitory activity. **Panel D** **–** The agonistic activity of VCN (FAK activation) was assessed in serum-starved non-migratory MDA-MB-435 cells kept in suspension and exposed to increasing concentrations of disintegrins for 30 min. Similar to dimeric CN, VCN is also shown to engage integrins agonistically (outside-in signaling).

By employing the recombinant system described above, two fusion proteins (Trx-rCN and Trx-VCN) were successfully expressed in the cytoplasm of Origami B (DE3) (see **Fig. 2A** for a comparison of Trx-VCN expression levels in different expression hosts; the data on Trx-rCN is not shown). Although the expression of Trx-VCN was robust in Origami B (DE3), by growing these transformants in a modified media recipe we have been able to boost the recombinant production level of VCN by at least one order of magnitude to a final expression yield of approximately 200 mg of active purified disintegrin per liter of bacterial culture.

A unique tobacco etch virus (TEV) protease cleavage site was engineered upstream of both disintegrin constructs in order to facilitate their subsequent cleavage from TrxA. TEV is a highly selective protease that recognizes with very high specificity the canonical Glu-Asn-Leu-Tyr-Phe-Gln-Gly amino acid sequence [43] therefore leaving the target recombinant proteins intact. This high specificity makes TEV an ideal molecular tool for processing recombinant proteins expressed as fusions. Moreover, TEV is a cysteine protease that relies on reducing equivalents (e.g., dithiothreitol) in order to be regenerated and act continuously. The addition of reducing equivalents during the purification process, however, could be detrimental to the integrity of any expressed recombinant proteins that rely on multiple disulfide bonds for activity. However, we found that the addition of exogenous reducing equivalents to Origami B lysates enriched in Trx-VCN or Trx-rCN was in fact not necessary for efficient proteolysis, since the bacterial lysates provided enough reducing equivalents for TEV regeneration. Therefore, the mere addition of a few units of recombinant TEV was sufficient to optimally process large quantities of expressed fusions (**Fig. 2B**). The released recombinant disintegrins were further processed and purified by reverse phase HPLC using a protocol originally designed for native disintegrins (see Materials and Methods).

### Initial evaluation of recombinant disintegrins for activity

The two purified recombinant molecules were initially tested for activity against platelets. Presumably, the main function of snake venom disintegrins in nature is to bind with very high affinity to the activated platelet αIIbβ3 integrin, thus efficiently inhibiting [20] the last step in the blood clotting, platelet aggregation, a process mediated by platelet integrin αIIbβ3. To our surprise, the platelet aggregation assay showed that only chimeric VCN retained full activity against activated αIIbβ3 integrin (with a calculated IC_50_ of ∼60 nM), whereas the rCN construct showed no activity in the nanomolar range characteristic of snake venom disintegrins (**Fig. 2C**). It appears that the latter construct, although soluble, had failed to fold correctly in the region where the binding site of the molecule resides (i.e., the 11-amino acid disintegrin loop).

### Mass spectrometry analysis and sequencing of VCN

Interestingly, the MS analysis (MALDI-TOF and ESI) demonstrated that, unlike native CN, VCN is a monomer (MW = 7146.0). The sequence was subsequently confirmed by tryptic MS sequencing. Based on these data, we speculated that although VCN folded correctly in the C-terminal half of the molecule, hence being a functional disintegrin, it may have adopted a non-native cysteine pairing in the N-terminal half of the molecule compared to CN [24] which compromised its dimerization (see **Fig. 1** for disulfide bond configuration of native CN).

### Focal adhesion kinase (FAK) phosphorylation studies

Native CN was previously shown to bind to integrins agonistically [34], [44], while acting as a potent soluble ECM-mimetic [35]. Interestingly, depending of the cellular context (adherent vs. suspended cells) CN was previously shown to alter the phosphorylation status of FAK (i.e., one of the earliest signaling events downstream of integrin engagement) by either activating (in serum-starved cancer cells kept in suspension in serum-free media and receiving no external input via integrins other than the disintegrin treatment) or deactivating (in adherent cells plated on various matrices) this non-receptor kinase [35]. In the present study we wanted to understand whether VCN: 1. retains the ability of native CN to signal via integrins agonistically in serum-starved non-migratory suspended cells receiving no external input other than disintegrins, and 2. has the same potency as CN by evoking this downstream signal in a dose-dependent manner. Our data suggest that indeed VCN also engages integrins agonistically (**Fig. 2D**), with a similar potency and in a dose-dependent manner comparable to native CN. These new data imply that the previously described signaling effects of CN [44] observed downstream of integrins in suspended cells were not in fact owing to CN's dimeric structure (i.e., signaling via receptor crosslinking), but may have rather been the result of an intrinsic property of the disintegrin fold, common to all disintegrins regardless of their tertiary structure.

### Cell surface binding analysis by flow cytometry

**Figure 3:**
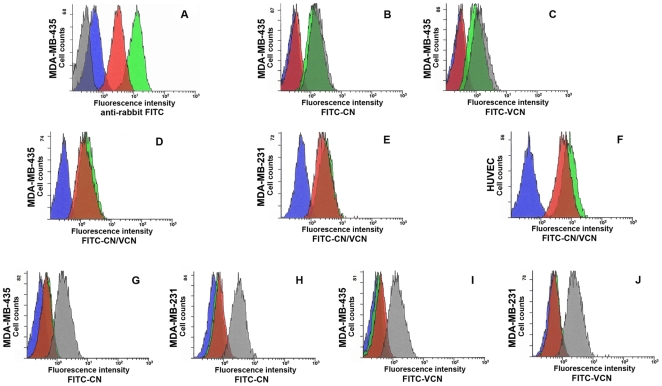
Binding analysis of FITC-labeled disintegrins by flow cytometry. **Panel A –** Both CN and VCN are detected at cell surface when probed with CN polyclonal antiserum. MDA-MB-435 cells were incubated with either VCN (red) or CN (green) followed by CN rabbit polyclonal antiserum and an anti-rabbit FITC-labeled secondary antibody. The controls included cells incubated with either anti-rabbit FITC-labeled secondary only (grey) or CN antiserum followed by the FITC-labeled secondary (blue). **Panels B, C** **–** FITC-CN (panel B) or FITC-VCN (panel C) fail to bind to cells prewashed in EDTA media, but once bound in regular media the subsequent addition of EDTA does not displace them from integrins. MDA-MB-435 cells were either incubated with FITC-labeled disintegrins only (grey) or with labeled disintegrins and then washed and resuspended in 3 mM EDTA media (green) or preincubated in 3 mM EDTA and then washed and exposed to labeled disintegrins (red) or just probed with an FITC-labeled irrelevant antibody control (blue). **Panels D, E and F –** Labeled disintegrins bind in a similar manner to cells with different integrin profiles. MDA-MB-435, MDA-MB-231 or HUVEC were either incubated with FITC-CN (green) or FITC-VCN (red) or probed with an FITC-labeled irrelevant antibody control (blue). **Panels G, H, I and J** **–** FITC-labeled disintegrins fail to bind to cells pretreated with either unlabeled disintegrins or an antibody competitor. MDA-MB-435 or MDA-MB-231 cells (panels G and H) were either incubated with FITC-CN only (grey) or an FITC-labeled irrelevant antibody control only (blue) or preincubated with unlabeled VCN (green) or 7E3 (red) and then probed with FITC-CN. Similarly, MDA-MB-435 or MDA-MB-231 cells (panels I and J) were either incubated with FITC-VCN only (grey) or an FITC-labeled irrelevant antibody control only (blue) or preincubated with unlabeled CN (green) or 7E3 (red) and then probed with FITC-VCN. The data are representative of four independent experiments.

The ability of VCN to mimic the binding behavior of native CN against different cell lines, as well as in the presence of EDTA or specific competitors, was tested by flow cytometry (**Fig. 3**). Our results show that FITC-labeled VCN has a similar binding profile to CN against HUVEC, MDA-MB-231 and MDA-MB-435 cells. These cells were chosen because they all express varying amounts of the RGD-dependent integrins αvβ3, αvβ5 and α5β1, the targets of CN, VCN and other ligands used in the study. We tested these cells by flow cytometry for cell surface integrin expression and found that there was a little difference in the relative amounts of integrins αvβ5 and α5β1 expressed by these cells, but a significant difference with respect to integrin αvβ3 expression with MDA-MB-435 expressing much higher amounts of this integrin compared to the other two cell lines, and MDA-MB-231 expressing the least amount. We also found that cell surface-bound VCN is recognized by rabbit polyclonal CN antiserum and bound VCN has the ability to block the binding of FITC-labeled CN, while a specific competitor, the 7E3 antibody raised against β3 integrin, is shown to block the binding of both FITC-labeled CN and VCN. Interestingly, regarding the latter observation, the converse appears to be also true for bound CN alone, Trx-VCN alone, or VCN plus polyclonal CN antiserum, since all these prevented the binding of 7E3 to the cell surface. However, VCN alone did not (data not shown), which led us to the conclusion that 7E3's binding site on β3 integrin [45] must map very closely to the ligand-recognition region for both disintegrins. As reported by Artoni et al. [45] 7E3's binding site was mapped between the C177-C184 loop and W129 residue in human β3 integrin subunit, in a region that is spatially close to the β3 subunit MIDAS (metal-ion dependent binding site), where the Asp residue of the RGD motif is also known to bind. Similar flow cytometry results were observed when the cyclic RGD peptide, cyclo(Arg-Gly-Asp-DPhe-Val) was used as a competitor instead of 7E3. When these cells were pre-incubated with micromolar concentrations of this cyclic RGD peptide before being exposed to fluorescently-labeled disintegrins, the cyclic RGD treatment also prevented the binding of both disintegrins. Unlike 7E3, the flow cytometry analysis showed that the αvβ3-specific monoclonal antibody LM609 [46] does not compete for the same binding region with either CN or VCN (data not shown). Finally, since the engagement of integrins by disintegrins was previously shown to be a metal-ion depended process [41], we asked whether a metal chelator such as EDTA will affect the binding of both CN and VCN. As expected, both CN and VCN did not bind to cells that were washed in 3 mM EDTA before being exposed to either disintegrin. However, once CN or VCN bound to the cell surface, the subsequent addition of EDTA does not appear to displace either disintegrin from their receptors (**Fig. 3**).

### Integrin binding kinetics by fluorescence polarization

To further determine the specific binding affinities of both native CN and VCN to purified (αvβ3 and αvβ5) or recombinant (α5β1) functional integrins, we measured these interactions in solution by fluorescence polarization. From this set of experiments, the dissociation constants for both native CN and VCN (**Table 1**) were deduced. These data showed that both disintegrins exhibit nearly identical affinities for αvβ3 and a similar affinity for αvβ5. The prediction that the sequence modification of VCN would alter its affinity for α5β1 compared to the native molecule was confirmed by this assay. The results demonstrated that there is at least an order of magnitude difference in these molecules' Kd values to α5β1 with VCN showing a higher binding affinity for this receptor (the average Kd values for integrin α5β1 were 15.2 nM for VCN, and 191.3 nM for native CN).

**Table 1:** Disintegrin-integrin biding kinetics by fluorescence polarization.

Disintegrin	Integrin Kd (+/−SD)		
	αvβ3	α5β1	αvβ5
CN	6.6 nM (0.8)	191.3 nM (65.2)	19.5 nM (5.7)
VCN	7.4 nM (0.4)	15.2 nM (4.2)	41.2 nM (12.3)

The binding kinetics were calculated from the fluorescence anisotropy data generated by the steady state binding of FITC-labeled disintegrins to either purified (αvβ3 and αvβ5) or recombinant (α5β1) functional human integrins. The dissociation constants for interactions of either CN or VCN with soluble integrins were determined by Scatchard analysis using a non-linear curve fit.

### Cell viability studies with adherent cells

**Figure 4:**
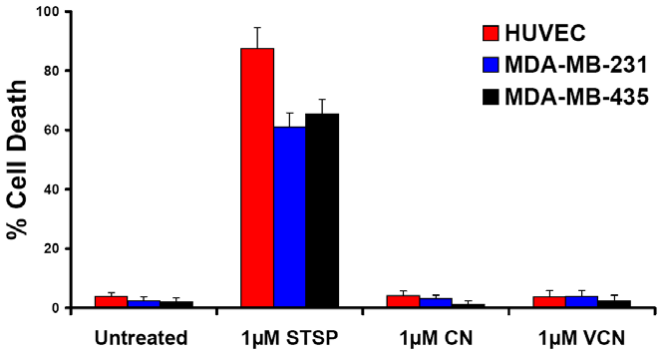
VCN does not affect the viability of cells plated on top of Matrigel. HUVEC, MDA-MB-231 or MDA-MB-435 cells were seeded in serum-free media in multiwell chamber slides on complete Matrigel and allowed to adhere for 1 hr. Once adherent, the cells were incubated for up to 48 hr with either CN or VCN up to a maximum concentration of 1 µM. Untreated cells or cells exposed to the apoptosis inducer Staurosporine (STSP) at a concentration of 1 µM were used as controls. The cells were fixed, TUNEL-stained and counterstained with Hoechst 33342. The amount of cell death was plotted for each condition by digitally counting the apoptosis events in random fields from images taken from multiple experiments for each condition.

To understand how VCN affects cell viability, we tested a range of CN and VCN concentrations (1–1000 nM) on HUVEC, MDA-MB-231 and MDA-MB-435 cells seeded on top of Matrigel (see Materials and methods) and compared the results to either untreated cells or cells exposed to Staurosporine, a known apoptosis inducer. Neither disintegrin showed any significant impact on cell viability regardless of the length of the incubation time (up to 48 hr) these cells were exposed to disintegrins. The cell viability was monitored using a MTS-based colorimetric assay and further confirmed by TUNEL staining (**Fig. 4**). In this later assay, at the end of the incubation period, the cells from all conditions were fixed in 4% formaldehyde, permeabilized in 0.2% Triton X-100, FITC-TUNEL stained, and then counterstained with Hoechst 33342. The amount of cell death was plotted for each condition by counting the apoptosis events in random fields from multiple independent experiments using the formula ‘number of apoptotic nuclei/total number of nuclei x 100’. Irrespective of the cells tested, neither CN nor VCN caused cell death under these conditions.

### Inhibition of cell invasion through a reconstituted cell membrane

**Figure 5:**
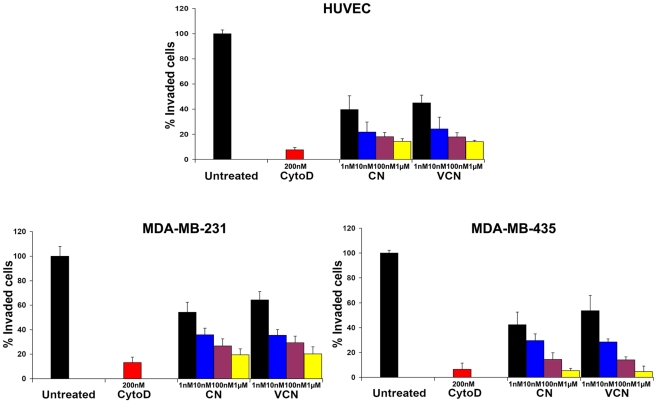
VCN inhibits cell invasion through a reconstituted basement membrane in a dose-dependent manner. The anti-invasive properties of VCN were tested in a modified Boyden chamber assay where serum-starved HUVEC, MDA-MB-231 or MDA-MB-435 cells were preincubated with various concentrations of disintegrins (1–1000 nM) for 10 min before being seeded into Matrigel-coated (ECMatrix™, Millipore) porous inserts (pore size, 8 µm) and allowed to invade against a chemoattractant gradient (HT1080 human fibrosarcoma conditioned media) for up to 48 hr. The fungal metabolite Cytochalasin D, a potent inhibitor of actin polymerization, was used as a positive control at a concentration of 200 nM. The above data were averaged from three independent experiments for each cell line tested.

The anti-invasive properties of VCN were tested *in vitro* using a modified Boyden chamber assay where serum-starved HUVEC, MDA-MB-231 or MDA-MB-435 cells were preincubated with various concentrations of disintegrins (1–1000 nM) for 10 min before being seeded into Matrigel-coated (ECMatrix™, Millipore) porous inserts (pore size, 8 µm) and allowed to invade against a chemoattractant gradient (HT1080 human fibrosarcoma conditioned media) for up to 48 hr (depending on the cell line). At the end of the incubation time, the cells that invaded into the lower chamber were detached, lysed, stained with CyQuant and quantitated in a fluorescent plate reader. The fungal metabolite Cytochalasin D, a potent inhibitor of actin polymerization, was used as a positive control at a concentration of 200 nM. Similar to native CN, VCN is shown to significantly inhibit HUVEC, MDA-MB-231 or MDA-MB-435 cell invasion in a dose-dependent manner (**Fig. 5**).

### Inhibition of HUVEC tube formation

**Figure 6:**
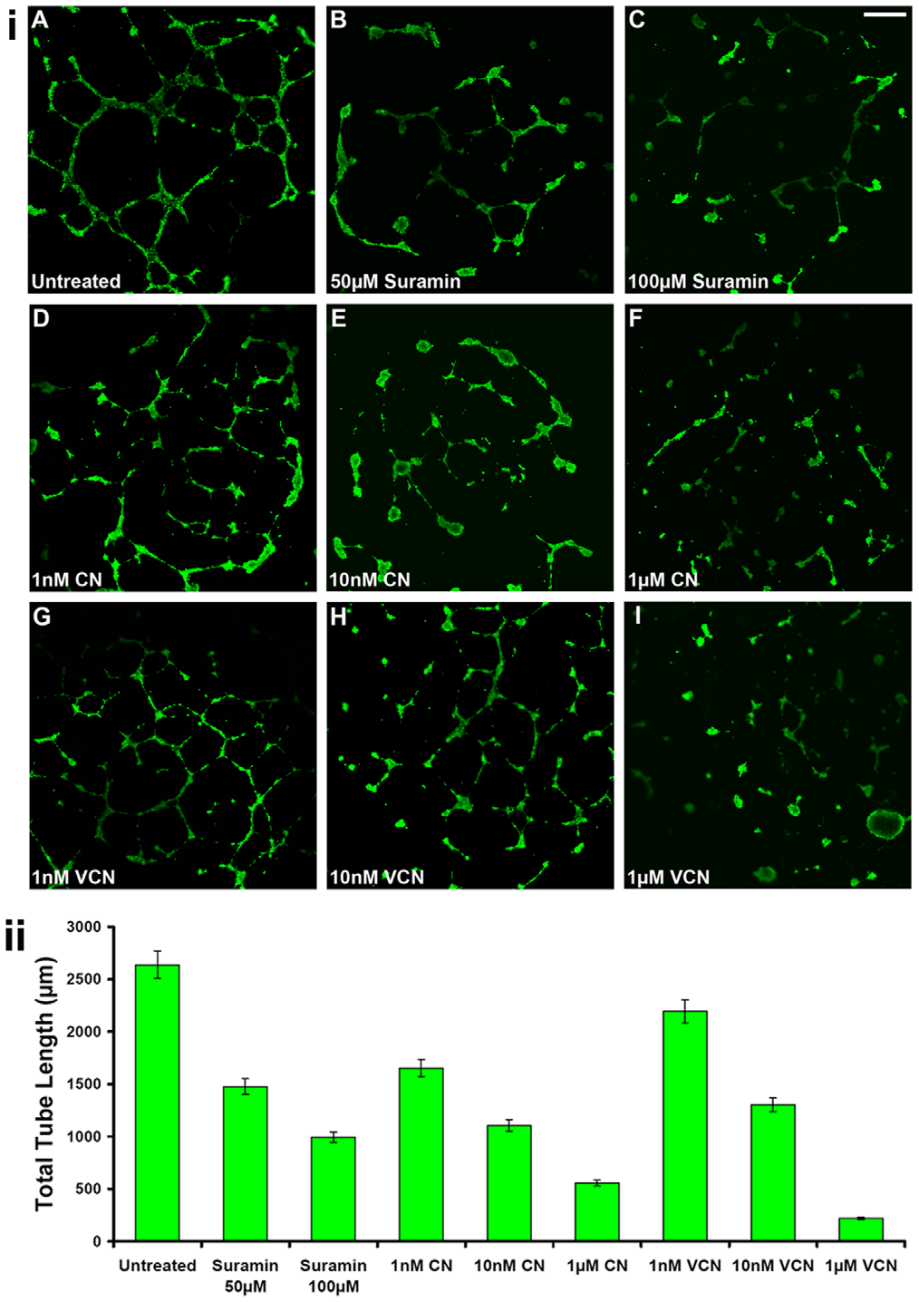
VCN inhibits HUVEC tube formation (tubulogenesis). (**i**) HUVEC were plated on ‘Endothelial Cell Tube Formation’ plates (BD Biosciences) in the presence of various concentrations of either CN or VCN (1–1000 nM) and allowed to form tubes after incubation for 12–16 hr at 37°C in the presence of 5%CO_2_. Suramin, a known tube formation inhibitor, was used as a positive control at two different concentrations (50 and 100 µM). At the end of the incubation period, cells were stained with Calcein AM and imaged by confocal microscopy (magnification, ×25). Representative figures from three independent experiments are shown above (scale bar, 200 µm). (**ii**) The degree of tubulogenesis was assessed by capturing multiple photomicrographs for all conditions on which the total length of the tubes was measured and computed in multiple fields using the Zeiss LSM Image Browser (Carl Zeiss MicroImaging GmbH) and then averaged to form each data point. The data presented above was assembled from three independent experiments.

The ability of VCN to inhibit HUVEC tubulogenesis was tested *in vitro* in an assay where HUVEC were plated on ‘Endothelial Cell Tube Formation’ plates (BD Biosciences) in the presence of various concentrations of either CN or VCN (1–1000 nM) and allowed to form tubes after incubation for 12–16 hr at 37°C in the presence of 5%CO_2_. In this experimental setting, Suramin, a known tube formation inhibitor, was used as a positive control at two different concentrations (50 and 100 µM). At the end of incubation period, the cells were stained with Calcein AM and imaged by confocal microscopy. As previously reported with native CN [47], VCN was also shown to potently inhibit HUVEC tube formation in a dose-dependent manner (**Fig. 6i**). In this assay, the tubes in each field were measured by three individuals in a blinded experiment and the total tube length averaged and plotted for each data set (**Fig. 6ii**).

### Disruption of HUVEC actin cytoskeleton organization

**Figure 7:**
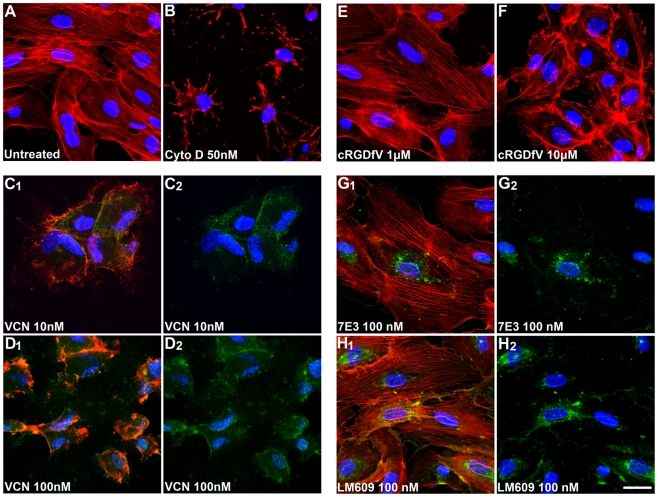
VCN induces massive actin cytoskeleton reorganization in HUVEC seeded on Matrigel. HUVEC were seeded in serum-free media in multiwell chamber slides on complete Matrigel, allowed to adhere, and then treated for 3 hr with various concentrations of cRGDfV peptide (1 and 10 µM) or FITC-VCN (10 and 100 nM) or the monoclonal antibodies 7E3 (100 nM) or LM609 (100 nM). When cells were incubated with integrin-binding antibodies (7E3 or LM609), a FITC-conjugated secondary antibody was used to track these treatments. The actin modifier Cytochalasin D was used as a positive control (50 nM). At the end of the incubation period, the cells from all conditions were fixed in 4% formaldehyde, permeabilized in 0.1% Triton X-100, stained with Rhodamine-Phalloidin and Hoechst 33342, and imaged by confocal microscopy. The cells exposed to FITC-labeled treatments (VCN or integrin-binding antibodies) are triple stained. The images shown above are Rhodamine-Hoechst only (panels A–B and E–F), FITC-Hoechst (panels C_2_-D_2_ and G_2_-H_2_) or overlayed three fluorophores (panels C_1_-D_1_ and G_1_-H_1_). Representative confocal images from multiple experiments taken at the same magnification (×630) are shown above (scale bar, 20 µm).

To assess the effect of VCN on cell morphology and actin cytoskeleton organization, HUVEC were allowed to adhere to complete Matrigel before being exposed to various treatments. Our results show that unlike other integrin ligands, including a small cyclic RGD peptide, and two integrin-binding antibodies (7E3 and LM609), VCN potently collapses the actin cytoskeleton of HUVEC in the low nanomolar range (**Fig. 7**). However, unlike VCN which exerts its maximal effects on the actin cytoskeleton of HUVEC in the low nanomolar range (10–100 nM), the cyclic RGD peptide used in the study appears to have some minor effects on the actin cytoskeleton of these cells (i.e., partial disassembly of stress fibers) only at a much higher concentration (10 µM). Moreover, the effect observed with VCN can be only partially prevented if the cells are preincubated with either the 7E3 antibody or the cyclic RGD peptide cRGDfV before being exposed to the recombinant disintegrin (data not shown). It is noteworthy that VCN does not detach HUVEC plated on Matrigel. Complete Matrigel is a tumorigenic matrix that was shown to be very similar in ECM composition to basement membranes (it is rich in collagen IV and laminins) and a number of collagen- and laminin-binding integrins (e.g., α1β1, α2β1, α3β1, α6β1, and α6β4) are employed by various cells to attach to Matrigel in a RGD-independent manner. On the other hand, VCN is an RGD-displaying polypeptide that was shown to bind to at least 4 RGD-recognizing integrins (i.e., αIIbβ3, αvβ3, αvβ5, and α5β1) but may, although not investigated yet, also bind to other RGD-dependent integrins. For instance, there are 8 human integrin members that have been described to recognize the RGD motif: all αv integrins (αvβ1, αvβ3, αvβ5, αvβ6, and αvβ8), αIIbβ3, α5β1, and α8β1. Because HUVEC do express collagen- and laminin-binding integrins that enable these cells to attach to Matrigel in a RGD-independent manner, it is not surprising that VCN does not detach HUVEC from Matrigel when these cells are exposed to the agent. However, the massive actin cytoskeleton reorganization induced by VCN in Matrigel-plated HUVEC raises the possibility that some of these effects might be relayed agonistically via one or a combination of integrins that VCN is know to bind to (i.e., transdominant integrin inhibition via either αvβ3, αvβ5, α5β1, or a combination of these receptors), integrins that are also expressed by these cells. Nonetheless, the observed signaling events induced by VCN in Matrigel-plated HUVEC need to be further dissected at the molecular level in order to better understand the complex integrin cross-talk associated with HUVEC adhesion, migration and invasion in tumorigenic matrices.

### Induction of apoptosis in tubulogenic HUVEC embedded in Matrigel

**Figure 8:**
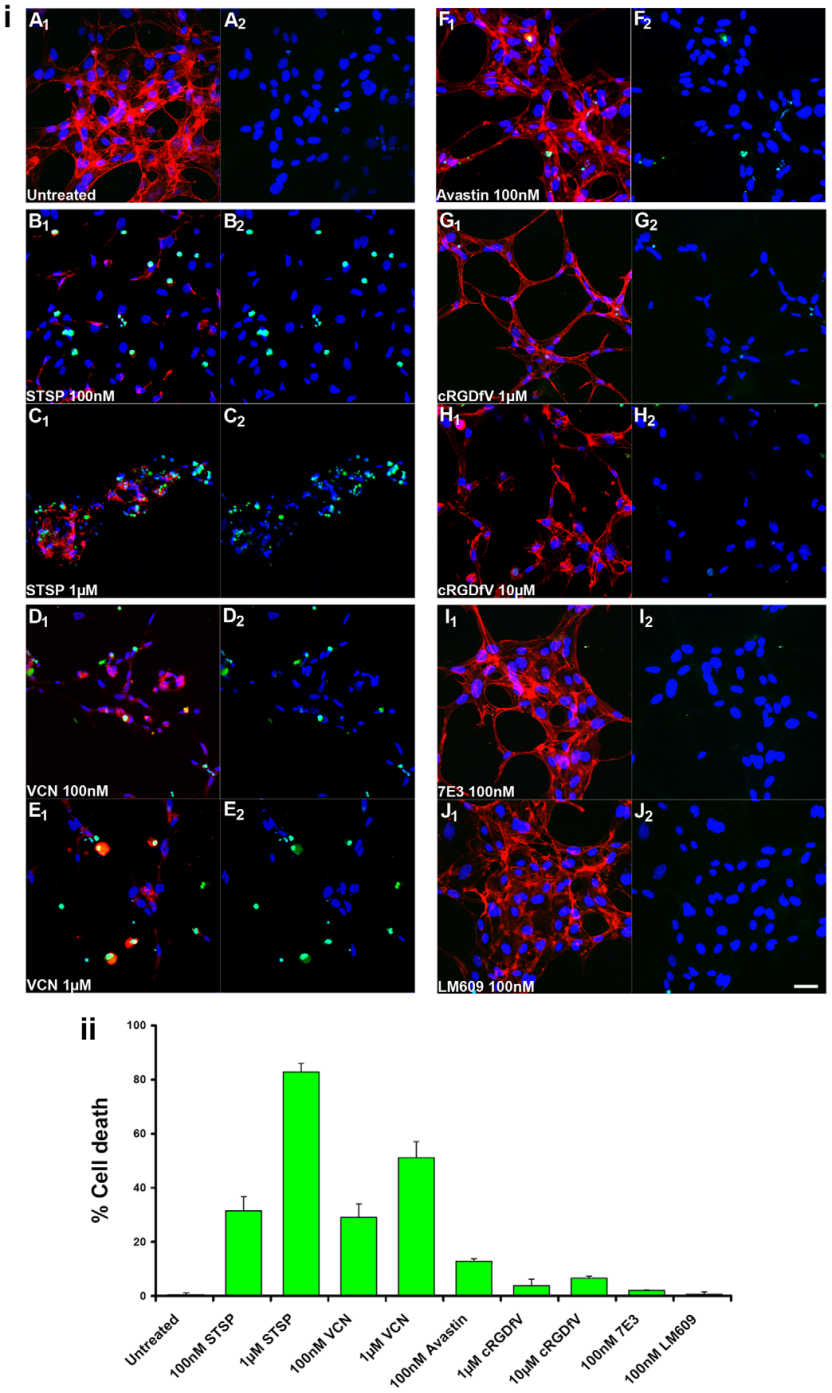
VCN induces apoptosis in tubulogenic HUVEC seeded between two Matrigel layers. HUVEC were seeded in serum-free media in multiwell chamber slides on complete Matrigel, allowed to adhere for 1 hr after which another layer of complete Matrigel was uniformly pipetted on top of the adherent cells. After another hour of incubation, different treatments were added to the media: either VCN (100 and 1000 nM), the cRGDfV peptide (1 and 10 µM), Avastin (100 nM), the β3 integrin 7E3 antibody (100 nM) or the αvβ3 integrin antibody LM609 (100 nM). Staurosporine (STSP), a known HUVEC apoptosis inducer and actin modifier, was used as a positive control at two different concentrations (100 and 1000 nM). The cells were then incubated for 16 hr at 37°C in the presence of 5%CO_2_. At the end of the incubation period, the cells from all conditions were fixed in 4% formaldehyde, permeabilized in 0.2% Triton X-100, FITC-TUNEL stained, and counterstained with Rhodamine-Phalloidin and Hoechst 33342. (**i**) Representative confocal images from multiple experiments taken at ×250 magnification are shown above (scale bar, 40 µm; panels A_1_-J_1_ –all fluorophores overlayed, panels A_2_-J_2_ - FITC-Hoechst). (**ii**) The amount of cell death was plotted for each condition by counting the apoptosis events from multiple random fields using the formula ‘number of apoptotic nuclei/total number of nuclei x 100’. The data shown above was generated from four independent experiments.

As stated above, VCN does not affect HUVEC viability if adherent cells plated on top of Matrigel are exposed to this agent, but it has significant anti-proliferative and anti-migratory effects (inhibition of tube formation) on these cells. Interestingly, when HUVEC are sandwiched between two layers of complete Matrigel, a significant apoptotic effect is also observed in the presence of VCN, but not with other integrin ligands (**Fig. 8i**). Surprisingly, a similar effect was also seen with Avastin in this setting, though less pronounced than with VCN. It is noteworthy that the cells sandwiched between two Matrigel layers were observed to migrate and form tubes much faster than HUVEC plated on top of Matrigel. The faster migrating cells sandwiched in Matrigel may rely more on the integrity of their focal adhesions coupled with a more dynamic actin cytoskeleton not only for migration, but also for survival. Thus, the profound effect induced by VCN on the actin cytoskeleton of these cells may explain the discrepancy seen in cell survival between the two experimental settings. It is also important to note that, unlike the two integrin binding antibodies, the cRGDfV peptide did alter the morphology of the tubes formed by HUVEC when sandwiched in Matrigel, although to a lesser extend than VCN and with no impact on cell viability. The amount of cell death was quantitated digitally (the ‘SimplePCI’ software) for each condition by counting the apoptosis events in multiple random fields from images taken from multiple experiments using the formula ‘number of apoptotic nuclei/total number of nuclei x 100’. The quantitation data (**Fig. 8ii**) was generated from four independent experiments.

### Liposomal encapsulation of VCN

Some theoretical advantages associated with liposomal encapsulation include: (i) enhanced drug delivery by increased tumor entrapment (passive targeting), (ii) prolonged drug half-life and thus reduced dosing frequency, and (iii) fewer drug-related toxicities. Our previous study showed that liposomal CN has: no immunogenicity, extended circulatory half-life, and undetectable non-target effects [22]. In the present study, batches of LVCN were prepared by sonication (LVCN-S) or homogenization (LVCN-H) using different processing conditions. Due to some favorable structural attributes characteristic of the disintegrin class of polypeptides (i.e., hydrophilicity, excellent stability in solution, at low pHs, in organic solvents, and to a range of temperatures), we found that CN and VCN can be readily encapsulated in liposomes with high efficiencies while retaining full biological activity. The encapsulation efficiency for these batches was 70% or greater (the average size of homogenized LVCN was 83 nm).

### *In vivo* efficacy studies with LVCN

**Figure 9:**
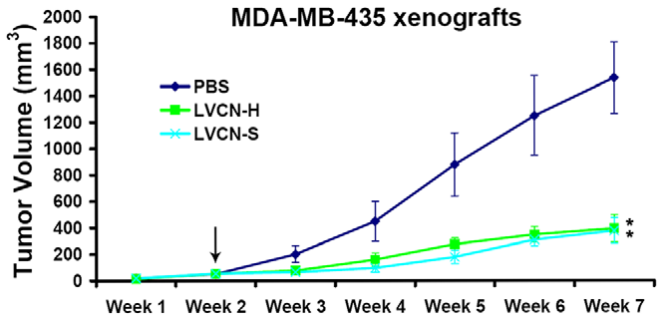
The initial evaluation of liposomal formulations of VCN in the MDA-MB-435 xenograft model. Nude mice were inoculated orthotopically (mammary fat pads; 5×10^5^ MDA-MB-435 cells in complete Matrigel per mouse) and tumors allowed to grow until they became palpable before the treatments were initiated (indicated by the arrow). The animals (n = 5) were treated twice a week with liposomal formulations of VCN that were prepared either by sonication (LVCN-S) or homogenization (LVCN-H). The latter method of encapsulation is suitable for scale-up production and was done in a microfluidizer. All LVCN-treated animals received the dose-equivalent of 100 µg of VCN per injection and were compared to a control group that received saline only. All treatments were administered intravenously twice a week via tail vein. The statistical analysis was done using ANOVA with Dunnett's *post-hoc* multiple comparison tests (* signifies a P<0.001). The liposomal formulations tested showed comparable efficacies. This animal model was repeated three times.

**Figure 10:**
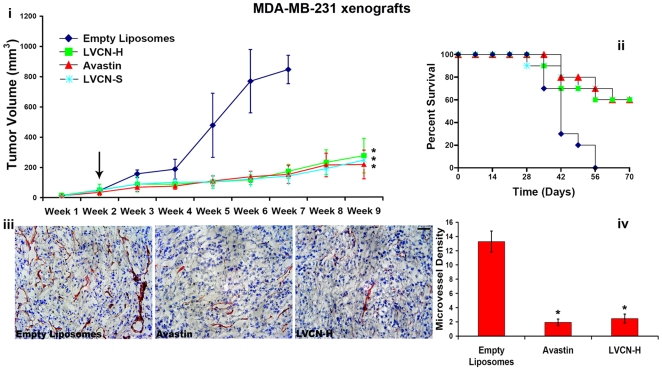
The efficacy and anti-angiogenic activity of LVCN in the breast MDA-MB-231 xenograft model. (**i**) Nude mice inoculated orthotopically (mammary fat pads; 2.5×10^6^ MDA-MB-231 cells in complete Matrigel per mouse) were allowed to grow palpable tumors before treatment was commenced (indicated by the arrow). The groups of animals (n = 10) were treated intravenously with either LVCN-H or LVCN-S (the dose-equivalent of 100 µg of VCN per injection) each administered twice a week, or Avastin (400 µg per injection; approx. 20 µg/gr) administered once a week. The control group received empty liposomes only. When compared to the control group, a significant delay in tumor growth was observed in all treated groups. (**ii**) The animal data shows increased survival in all treated groups compared to the control group (all control animals died by the end of week 7) (**iii**) Tumor cryostat sections from all groups were fixed in acetone, stained with a polyclonal CD31 antibody (clone MEC13.3) and counterstained with hematoxylin. Representative CD31 photomicrographs are shown above (scale bar, 100 µm). (**iv**) For microvessel quantitation, random CD31-positive areas from multiple fields were counted using a computer-assisted method (the ‘SimplePCI’ imaging software) and plotted as % of total stained area. Similar to Avastin, our data indicates that VCN exhibits potent anti-angiogenic activity in this animal model. ANOVA was used for statistical analysis followed by Dunnett's multiple comparison tests (* signifies a P<0.001).

The initial efficacy evaluation of LVCN formulations was done in the MDA-MB-435 animal model [48]. Although the breast origin of this human cell line is controversial and currently under scrutiny [49], [50], these cancer cells are a high integrin αvβ3 expressor and thus constitute a good model for screening pharmacological inhibitors directed at this receptor. In the MDA-MB-435 model, nude mice were inoculated orthotopically (mammary fat pads; 5×10^5^ MDA-MB-435 cells in complete Matrigel per mouse) and tumors allowed to grow until they became palpable before the treatments were initiated. The animals (n = 5) were treated twice a week with liposomal formulations of VCN that were prepared either by sonication (LVCN-S) or homogenization (LVCN-H). Animals receiving saline or unencapsulated VCN (at the dose of 100 µg per injection administered twice weekly intravenously via tail vein) were used as controls. In this animal model both LVCN formulations showed good tumor growth inhibition efficacy. The model was repeated three times and the overall effect on tumor growth inhibition quantitated (**Fig. 9**). LVCN was further tested in the MDA-MB-231 breast carcinoma model with similar results (**Fig. 10**). In the latter model, LVCN was compared to Avastin by looking at several parameters: tumor growth inhibition efficacy, animal survival and reduction in microvessel density. Avastin (bevacizumab), is a monoclonal antibody that interferes with neo-vessel formation in tumors by trapping an essential growth factor for angiogenesis, VEGF-A (vascular endothelial growth factor A), currently representing the gold standard for antiagiogenesis therapy, being approved by the FDA for the treatment of several types of solid cancers [51]. In the MDA-MB-231 model nude mice inoculated orthotopically (mammary fat pads; 2.5×10^6^ MDA-MB-231 cells in complete Matrigel per mouse) were allowed to grow palpable tumors before treatment was initiated. The groups of animals (n = 10) were treated intravenously with either LVCN-H or LVCN-S (the dose-equivalent of 100 µg of VCN per injection) each administered twice a week, or Avastin (400 µg per injection; approx. 20 µg/gr) administered once a week. The control group received empty liposomes only. Another control group received unencapsulated VCN (at the dose of 100 µg per injection administered twice weekly intravenously via tail vein). When compared to the empty liposomes group, a significant delay in tumor growth was observed in all treatment groups. More importantly, in this model LVCN was found to significantly increase the survival of the animals similar to Avastin (all control animals in this model died by the end of week 7). As mentioned, both animal models included a control group that received unencapsulated VCN. Interestingly, although the direct injection of unencapsulated VCN at the above dose appeared to be well tolerated by the animals for the duration of the study, in neither model was there a significant therapeutic response as compared to the LVCN group (data not shown).

### *In vivo* evaluation of LVCN for anti-angiogenic activity

Tumor specimens from the MDA-MB-231 model were prepared for CD31 staining and microvessels quantitated as described in the ‘Materials and methods’ section. Tumor cryostat sections from all groups were fixed in acetone, stained with a polyclonal CD31 antibody (clone MEC13.3) and counterstained with hematoxylin. For microvessel quantitation, random CD31-positive areas in multiple fields on sections from multiple tumors were counted using a computer-assisted method (the ‘SimplePCI’ imaging software) and plotted as % of total stained area. Our data shows a dramatic reduction (>80%) in microvessel density in the LVCN group compared to the empty liposome control, and similar to the Avastin-treated group (**Fig 10**).

### *In vivo* evaluation of LVCN for pro-apoptotic and anti-proliferative activities

**Figure 11:**
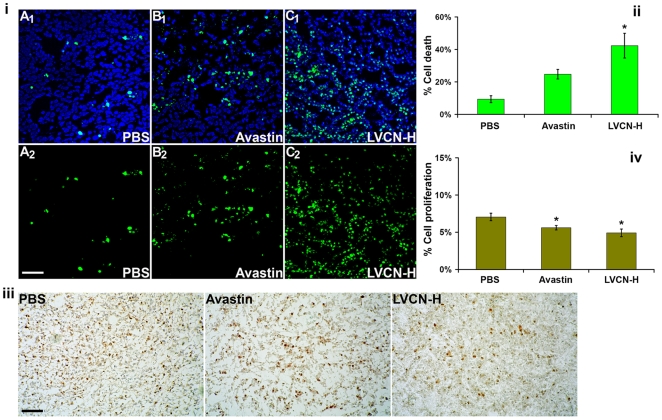
LVCN treatment shows enhanced tumor apoptosis in the breast MDA-MB-231 xenograft model. For this experiment, MDA-MB-231 xenografts were allowed to grow to a significantly larger volume (4 weeks after inoculation) before treatments were initiated. The animals received either liposomal VCN (at the dose-equivalent of 100 µg of VCN per injection) or Avastin (400 µg per injection) administered intravenously every other day and compared to a control group that received saline only. All animals were sacrificed after receiving 6 consecutive doses of each treatment. (**i**) To assess the impact of VCN on cell death, tumor cryostat sections from each group were stained with FITC-TUNEL, and counterstained with Hoechst 33342. Representative confocal images from multiple experiments taken at ×250 magnification are shown above (scale bar, 100 µm; panels A_1_-C_1_ - TUNEL-Hoechst, panels A_2_-C_2_ - TUNEL only). (**ii**) The amount of cell death was quantitated as ‘number of TUNEL^+^ nuclei/total number of nuclei x 100’ by counting all nuclei in ‘hotspot’ areas from multiple fields using a computer-assisted approach (the ‘SimplePCI’ imaging software). The liposomal VCN group shows a significantly increased amount of cell death compared to either Avastin or control. (**iii**) The impact of VCN treatment on tumor proliferation was assessed by Ki-67 immunoperoxidase staining. Representative Ki-67 images are shown above (scale bar, 200 µm). (**iv**) Cell proliferation was quantitated using the same approach as for TUNEL staining. The differences in cell proliferation between the treatment groups were much smaller than those observed for cell death. The data was analyzed with ANOVA followed by *post-hoc* tests (* signifies a P<0.01).

The effect of LVCN on tumor cell death or proliferation was also evaluated in the MDA-MB-231 model. Unlike the efficacy study, in this study tumors were allowed to become more established (4 weeks from inoculation) before a short course of treatment (6 doses) with either LVCN or Avastin was initiated. Our results show that there was a large difference in the amount of cell death (TUNEL staining) between either LVCN- or Avastin-treated groups and the control. As mentioned above, the difference in tumor proliferation was assessed by Ki-67 staining, a commonly employed proliferation marker with prognostic value in various human malignancies including breast cancer [52], [53]. Although there were statistically significant differences in the amount of Ki-67 staining between the groups, the LVCN group showing the least amount of staining, these differences were much smaller than the ones observed for cell death (**Fig. 11**). We chose to quantitate the effects of various treatments on tumor apoptosis and proliferation in more established tumors that received only a short course of therapy rather than in tumors harvested at the end of the efficacy study (after 7 weeks of treatment). The reason for this decision was because in the study shown in **Fig. 10**, despite the significant differences found in tumor size and animal survival between the treated groups and control, we did not see similar differences in tumor apoptosis nor in proliferation possibly due to the ability of both LVCN and Avastin to induce tumor stabilization/dormancy after repeated administration over a longer period of time (data not shown).

## Discussion

Tumor angiogenesis involves a still poorly understood cross-talk between transformed epithelial cells and quiescent endothelial cells originating from preexisting vessels. In this process, events associated with a complex deposition of a new ECM in conjunction with multiple paracrine and autocrine loops are required to be precisely coordinated spatiotemporally for the successful execution of a specialized migratory program activated in quiescent endothelial cells [54]. As key regulators of cell migration, integrins function as centripetal signaling platforms or functional hubs [55] that bi-directionally integrate signaling circuitries downstream of different classes of cell surface receptors (e.g., the semaphorin/plexin/neuropilin, the growth factor/receptor tyrosine kinase and the cell surface protease systems) with the cellular locomotor apparatus [56]. In angiogenesis, integrins are known to regulate many cell decisions associated with this program by integrating the cellular viability pathways with various processes ranging from ECM deposition and degradation, directional endothelial cell migration and assembly into primitive cords, to lumen formation and vessel maturation [57], [58]. The precise spatial distribution of integrin-binding motifs encoded in normal and oncogenic variants of ECM polymers [59] is likely to represent the most important organizing principle of the dynamics of focal adhesion complexes assembled across plasma membranes, which coordinate via integrins the motility in both angiogenic endothelial and metastatic cancer cells. The efficient disruption of various integrin-mediated interactions formed between tumorigenic ECMs and angiogenic EC in tumoral microenvironments seems to be critical from the therapeutic standpoint since, as recently reported [60], one important downside of pharmacologic VEGF/PDGF blockade is the persistence of basement membranes from involuted tumor vessels after both EC and pericytes undergo regression. This neo-vascular ECM that is left behind in the course of anti-angiogenesis therapy is suspected to provide a critical scaffold leading to a rapid repopulation of these ECM ‘tracks’ by new EC once the anti-VEGF/PDGF treatment is discontinued, in a process in which integrins probably play a major role.

The critical involvement of integrins in both angiogenesis [55], [61] and tumor metastasis [6] provides the rationale for developing therapeutic antagonists aimed at disrupting these molecularly intertwined processes [14]. Nonetheless, the development of efficacious integrin-targeted anti-cancer agents is complicated by the fact that multiple members of the integrin family appear to be differentially involved in distinct phases of tumor angiogenesis [55] and possibly metastasis, and a clear understanding of what combination of integrins is optimally required to be simultaneously targeted in order to efficiently disrupt these processes is still lacking. For instance, most efforts in the past were channeled at developing pharmacological agents directed at the RGD-binding alphav integrin members, a subclass of integrins thought to play pivotal roles in the regulation of pathological angiogenesis. These efforts led to the development of small RGD-mimetics, cyclic RGD peptides, and integrin-targeting monoclonal antibodies [15], [62]. The striking discrepancy that had been noticed to exist, however, between the genetic and pharmacologic models of alphav integrin ablation [10] prompted a reevaluation of these receptors as regulators of angiogenesis [63] and attempted to explain some of the disappointing results generated with alphav integrin targeting agents in clinical trials. The fact that mice deficient in either β3 or β5 or both β3 and β5 integrins not only develop normally, but paradoxically show an enhanced tumor angiogenic response [10] when challenged postpartum supports the idea that these two integrins might act in a more complex way than originally thought as regulators of pathological angiogenesis, and may be endowed with an unexpected tumor suppression function. Moreover, a tumor suppression function for alphav integrins appears to also exist in epithelial cells since the genetic ablation of alphav integrin gene in epithelial cells of murine skin leads to development of squamous cell carcinomas [64]. In order to reconcile these contradictory observations regarding the contextual role of alphav integrins alternative hypotheses must be explored. It is conceivable, for instance, that while the overexpression of alphav integrins in angiogenic endothelial cells may be an important requirement in regulating the migration of these cells on solid ECM support (tethered migration) which leads to their assembly into neovessels (a net pro-angiogenic effect), in the presence of monovalent soluble ECM fragments a cascade of inappropriate signals might be initiated and relayed through the same alphav receptors (in the absence of tethering) which ultimately may lead to cessation of cell migration and a dramatic reorganization of actin cytoskeleton in cells exposed to such ligands (a net anti-angiogenic effect). Although in the course of pathological angiogenesis these two mechanisms might coexist, in the tumor microenvironment the abundance of pro-angiogenic circuitries, driven by a continuous deposition of new ECM polymers, appears to render the anti-angiogenic signals ineffectual thus favoring a perpetual state of angiogenesis. Even though other members in the integrin family have also been linked to different forms of angiogenesis (in development vs. adult life, physiological vs. pathological), we speculate that the alphav members may play critical roles in promoting and, possibly more importantly, terminating the cell migration events associated with angiogenesis in adult life. This may help explain the inconsistency in outcomes between genetic and pharmacological models of alphav integrin ablation, providing a rationale for why animals devoid of β3 and β5 integrins display increased pathological angiogenesis postpartum. A number of endogenous ECM-derived fragments with anti-angiogenic activity have been characterized to date [17], [65], and some of these promising molecules were found to bind to either one or multiple integrin receptors (i.e., endostatin, tumstatin, arresten, canstatin, PEX, endorepellin etc) [66]. It is noteworthy that the integrin ligation by some of these endogenous fragments (i.e., endostatin, endorepellin) was shown to lead to the collapse of actin cytoskeleton and focal adhesion disassembly in endothelial cells *in vitro* [32], [33]. Interestingly, these cell motility deactivation effects observed in angiogenic endothelial cells with ECM fragments are reminiscent of those triggered by members [67], [68] belonging to an unrelated class of signaling molecules (i.e., the semaphorins) which suggest that a high degree of integration may exist between the semaphorin-plexin-neuropilin and integrin systems in controlling the actin cytoskeleton dynamics during angiogenesis [57]. It could be argued that, if integrins physically connect the actin cytoskeleton with a plethora of complex molecules bound across plasma membrane, thus enabling the dynamic actin cytoskeleton in migratory cells to sense their extracellular microenvironment, then a pharmacological agent designed to efficiently disrupt integrins is also expected to induce a massive reorganization or collapse of actin cytoskeleton in these cells. Since some of the endogenous ECM-derived anti-angiogenic fragments have already been characterized as potent tumor suppressors in various animal models, the biggest stumbling block to their successful clinical translation remained the ability to produce them recombinantly in large scale. However, this proved to be more complicated than originally thought due to the fact that these ECM fragments are naturally derived through proteolytic cleavage from higher molecular weight matrix polymers which makes them dependent on their parental supermolecules for correct folding (i.e., intramolecular chaperoning). Because of this, these otherwise promising molecules proved to be refractory to correct folding when expressed in various recombinant systems, which led to altered or diminished biological activity for the recombinant versions when compared to the native ECM fragments, an issue that may have accounted for the disappointing performance seen with recombinant endostatin in clinical trials [66].

Interestingly, several classes of metalloproteases found in snake venoms also contain domains that share significant structural similarities with modules and domains buried in ECM proteins [69]. Among these, the disintegrin and disintegrin-like domains found in snake venoms display a variety of integrin-binding motifs with enormous pharmacological potential. Similar to the anti-angiogenic fragments derived from mammalian ECMs, disintegrin are also generated through proteolytic cleavage from larger multidomain metalloproteases [70].

In this study, we show that a chimeric disintegrin, vicrostatin (VCN), derived from a member of a well characterized family of naturally occurring broad-spectrum integrin inhibitors, could be successfully produced recombinantly in large scale in an engineered bacterial system. The recombinant production of VCN was a complex achievement complicated by the fact that disintegrins are small polypeptides with almost no secondary structure that depend for their proper folding and biological activity on the correct pairing of a large number of disulfide bridges (5 in VCN) relative to their molecular size. For this reason, and because disintegrins do not express well in mammalian cells or yeast, a bacterial system supportive of disulfide bridge formation needed to be identified. We found that VCN can be expressed in such a system (e.g., Origami B) which prompted us to further optimize the expression method for this recombinant polypeptide in this system. Our optimization efforts led to consistent expression levels for this recombinant molecule in Origami B with minimal batch-to-batch variation and with yields around 200 mg of purified active VCN per liter of bacterial culture. Recombinant VCN is a synthetic construct that retains the RGD integrin-recognition motif displayed by the native disintegrin contortrostatin (CN) it was derived from, but was also engineered to exhibit some novel integrin biding characteristics. Therefore, in a number of *in vitro* functional assays, recombinant VCN was found to retain the binding properties of native CN, while showing an improved binding affinity compared to native CN for one important receptor in angiogenesis, integrin α5β1. The binding affinity of VCN for integrin α5β1 was measured by fluorescence polarization and found to be one order of magnitude higher than that of CN (Kd = 15.2 nM for VCN vs. 191.3 nM for native CN). The recombinant VCN behaves like a true disintegrin in that it inhibits platelet aggregation (by disrupting fibrinogen binding to integrin αIIbβ3) in a similar manner to native CN and with a almost identical IC_50_ (approx. 60 nM). Mass spectrometry analysis of VCN showed that this recombinant disintegrin is, unlike CN, a monomer which led us to speculate that VCN may have folded differently than CN in the N-terminal half of the molecule and this prevented its dimerization. Cell surface binding analyses by flow cytometry conducted with fluorescently-labeled disintegrins showed that VCN binds similarly to CN to different cell lines and, like CN, its cell surface binding is abolished in the presence of integrin ligands (either a cyclic RGD peptide or an antibody fragment) competing for the same binding sites. Since disintegrin binding is a metal-ion dependent process, we also showed that in the presence of metal chelators both CN and VCN cease to bind to cells. The ability of VCN to interfere with cell migration and invasion was further tested *in vitro* in two experimental settings: in a modified Boyden chamber using different cell lines (the transwell invasion assay) and against HUVEC in the tube formation assay. In all instances VCN was found to significantly inhibit cell migration and invasion in the nanomolar range and with a potency similar to that of native CN. Moreover, our previous studies indicated that disintegrins are not cytotoxic to cells cultured in normal conditions. In order to understand whether the anti-migratory/anti-invasive effects observed with VCN were due to its ability to interfere with essential components of cellular locomotor apparatus (i.e., the dynamic actin cytoskeleton of migratory cells) and was not the result of a cytotoxic effect, we conducted cell viability studies with both CN and VCN using HUVEC and cancer cells. Our results showed that both CN and VCN were completely devoid of cytotoxicity at all concentrations tested (up to 1 µM). Surprisingly, however, when HUVEC were sandwiched between two Matrigel layers, in an experimental setting that allows these cells to move faster and form tubes more rapidly, VCN did show a significant cytotoxic effect compared to other integrin-targeting ligands tested (a cyclic RGD peptide and two different antibodies). This effect was unexpected and suggests that rapidly migratory and/or invasive cells might be the most vulnerable to the effects of this agent. Our previous studies with native CN indicate that this disintegrin may behave like a soluble ECM-mimetic, potently altering the actin cytoskeleton dynamics by deactivating key molecular components of focal adhesions in both adherent HUVEC and glioma cells, the result of which is a net anti-migratory effect [35]. Like native CN and unlike other integrin-targeting ligands, we showed that VCN has a unique integrin binding profile (simultaneously targeting integrins αvβ3, αvβ5, and α5β1) and is able to induce at nanomolar concentrations the disassembly of actin stress fibers and a massive actin cytoskeleton reorganization in HUVEC plated on Matrigel. The ability of disintegrins to elicit an inappropriate signaling and alter the tensional homeostasis in angiogenic and cancer cells alike may have profound therapeutic implications since the characteristic increase in tissue rigidity associated with cancers was shown to critically modulate the behavior of malignant cells by regulating the ability of these cells to form focal adhesions and efficiently signal through growth factor receptors [71]. Furthermore, the ability to manipulate pharmacologically the actin cytoskeleton and stress fiber assembly in transformed cells may represent a novel therapeutic goal towards achieving tumor dormancy [72], [73]. Interestingly, the cytoskeletal effects observed with VCN in tubulogenic EC cultured in a rich tumorigenic matrix that is uniquely endowed to support both cell survival and migration are strikingly similar to those exerted by the endogenous ECM-derived fragments discussed above. As already mentioned, these effects distinguish VCN from the other integrin ligands that were tested in the same setting, including the small cyclic RGD peptide cyclo(Arg-Gly-Asp-DPhe-Val). It is noteworthy to emphasize that a methylated variant of the cyclic RGD peptide used in our experiments, the cyclo(Arg-Gly-Asp-DPhe-NMeVal) peptide or Cilengitide, which displays an improved specificity to integrins αvβ3 and αvβ5 [74], [75], has been evaluated in a number of advanced solid tumors in several clinical trials and showed promise against glioblastoma multiforme [76], [77]. Moreover, the two monoclonal antibodies that were included in our *in vitro* assays, the integrin αvβ3-targeting 7E3 and LM609, were also previously tested *in vivo* in a number of animal cancer models and reported to have good tumor growth inhibition efficacy [46], [78]. By comparing different integrin ligands, our *in vitro* data seem to indicate that molecules that behave like soluble ECM-mimetics might have additional anti-angiogenic benefits compared to cyclic RGD peptides and integrin-targeting monoclonal antibodies. Furthermore, our results also suggest that, unlike cyclic RGD peptides, VCN may produce effective anti-angiogenic effects at much lower doses which may possibly translate into fewer side effects.

Although VCN retains native CN's ability to bind the activated αIIbβ3 platelet integrin, like CN [22] it does not interact with quiescent platelets, an *in vitro* observation further corroborated by our *in vivo* findings: no side effects were documented following direct intravenous administration of purified VCN in two different species: up to 1 mg per shot in mice or up to 5 mg per shot in rats. It is important to mention that there is an established link between activated platelets and metastasis [79], [80] and from the therapeutic standpoint it may be advantageous to use a polypeptide like VCN that theoretically might also have the potential to address this metastasis strategy. As a small polypeptide, VCN is not expected to be immunogenic, and our preliminary animal studies showed that VCN indeed failed to elicit an antibody response following intravenous infusion. Despite these findings, for enhanced passive targeting of the drug to the tumor site, we opted for liposomal delivery. VCN can be efficiently encapsulated into unilamellar liposomes, unlike other proteins possibly due to its structural characteristics, and our findings indicate that the liposomal formulations of VCN have far superior efficacy compared to the naked polypeptide in two animal models of human cancer. In these models LVCN showed excellent tumor growth inhibition efficacy, increased animal survival and a significant reduction (>80% compared to control) in tumor microvessel density producing similar results compared to Avastin (bevacizumab). Interestingly, in a separate study of the same breast cancer model (MDA-MB-231 xenografts) VCN showed superior tumor apoptosis effects compared to Avastin as indicated by the quantitation of the TUNEL-stained areas on tumor sections. As already mentioned, in all these models the liposomal delivery of VCN was far more efficacious compared to the intravenous administration of the naked polypeptide. We do not exclude, however, the therapeutic scenario in which the naked polypeptide administered at much higher doses than those used in this study might show improved therapeutic responses. On the other hand, the liposomal delivery has a number of other advantages showing good therapeutic responses at lower drug doses and less frequent administrations. To better understand how VCN is released from liposomes in the tumor microenvironment, we are currently conducting mechanistic studies aimed at elucidating the fate of LVCN in tumor-bearing animals. As a unique broad-spectrum anti-invasive drug, VCN may hold an advantage over other anti-tumor therapeutic modalities in that it may be better suited to address the cell survival loops operating in the avascular tissue in the early steps of angiogenesis and metastasis [81], [82]. For instance, in an elegant animal study, Carbonell *et al*. [83] recently demonstrated the critical importance of interactions between the β1 integrin receptors on tumor cells and the vascular basement membrane in maintaining the viability of early metastatic seeds in the brain before the angiogenesis process is initiated. Moreover, recent studies have demonstrated that the use of various anti-VEGF/PDGF strategies is linked to an increased risk of early metastasis in animal cancer models [84], [85]. Although the clinical relevance of these preclinical studies is not yet clear [86], these data support the idea that not only is there an imperative need to design novel anti-angiogenic drugs with better anti-invasive properties, but also to test the impact of established anti-angiogenics (such as Avastin) on metastasis and/or postoperative survival when administered in combination with anti-invasive modalities. In our previous studies conducted with native CN we showed that this agent was very effective at blocking metastasis in an animal model [87]. To better understand the ability of VCN to function as an anti-metastatic agent, we are currently testing this agent in several spontaneous models of breast cancer metastasis (i.e., the murine 4T1 and D2F2, and human MDA-MB-231). In summary, we believe that the novel agent described in this report holds a promising translational potential and has a design advantage over endogenous anti-angiogenic fragments in that it can be made recombinantly in large quantities, safely infused into animals, and efficiently encapsulated into liposomes for enhanced tumor delivery.
